# C-X-C motif chemokine ligand 12 (CXCL12)/C-X-C motif chemokine receptor 7(CXCR7) regulates epithelial-mesenchymal transition process and promotes the metastasis of esophageal cancer by activating signal transducer and activator of transcription 3 (STAT3) pathway

**DOI:** 10.1080/21655979.2022.2048984

**Published:** 2022-03-09

**Authors:** Jing Guo, Chang-Yong Tong, Jian-Guang Shi, Xin-Jian Li

**Affiliations:** Department of Thoracic Surgery, Ningbo First Hospital, Ningbo, Zhejiang province, China

**Keywords:** Esophageal cancer, CXCR7, CXCL12, epithelial-mesenchymal transition, tumor metastasis

## Abstract

Esophageal cancer is a malignant tumor of the digestive system that is prone to metastasis. Chemokines and their receptors act an essential role in the occurrence and development of tumors. Here, we investigated the regulatory mechanism of CXCL12/CXCR7 in the growth and metastasis of esophageal cancer. CXCR7 was found highly expressed in clinical tissues and cells of esophageal cancer. Knockdown of CXCR7 inhibited the proliferation, migration, invasion, and epithelial-mesenchymal transition (EMT) process of esophageal cancer cells. The knockdown of chemokine CXCL12 also inhibited the expression of EMT-related proteins and the mesenchymal morphology changes of esophageal cancer cells, but the knockdown of C-X-C motif chemokine receptor 4 (CXCR4) had no such effect. Furthermore, the knockdown of CXCR7 attenuated the enhanced EMT process induced by CXCL12 overexpression, while the knockdown of CXCR4 cannot inhibit this process. In addition, overexpressed CXCL12/CXCR7 activated the downstream STAT3 pathway, but had little effect on the extracellular regulated protein kinase (ERK) or serine-threonine kinase (AKT) pathways. Inhibition of the STAT3 pathway using AZD9150 weakened the accelerated effects of CXCL12/CXCR7 on the growth and metastasis of esophageal cancer in vitro and in vivo. In conclusion, our research revealed that CXCL12/CXCR7 regulates EMT and other malignant processes by activating the STAT3 pathway to accelerate the growth and metastasis of esophageal cancer.

## Graphical Abstract

**Figure:**
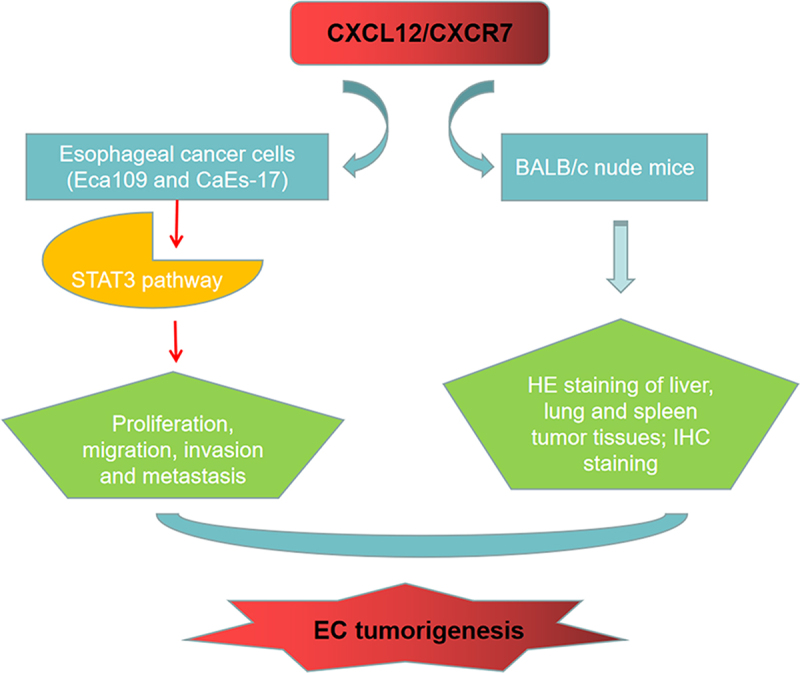

## Introduction

Esophageal cancer (EC) is a malignant tumor with the eighth highest incidence rate and sixth highest mortality rate in the world according to the global cancer statistics in 2020, and it is a serious threat to human life and health [1]. The onset of EC is relatively inconspicuous, most patients are asymptomatic at the early stage and more than half of patients have entered the middle or advanced stages when they seek medical treatment [2]. The treatment of EC is mainly depend on surgery combined with radiotherapy, chemotherapy, targeted and immunotherapy [3]. Although the existing treatments are constantly updated and have made great breakthroughs, the overall five-year survival rate of EC patients is still less than 30% [4]. Postoperative recurrence and metastasis are major factors affecting the prognosis of patients [5]. EC cells are prone to invade the submucosa in the early stage, and infiltrate and metastasize through the mucosa and the extensive lymphatic and vascular network under the mucosa [6]. Therefore, identifying effective molecular targets to inhibit the recurrence and metastasis of EC is essential for the treatment of EC.

The metastasis of tumor is a complex process, and epithelial-mesenchymal transition (EMT) is considered to be one of the key steps for the invasion and metastasis of epithelial-derived malignant tumors [7]. Normally, there are tight junctions and adherents between epithelial cells and between epithelial and stromal cells that maintain cell morphology and support the cytoskeleton [8]. However, when epithelial phenotype cells are stimulated and transformed into mesenchymal phenotype through a series of pathways, the epithelial cells will gradually lose their cobblestone-like morphology and change to a spindle-like mesenchymal morphology [8,9]. At the same time, epithelial cells exhibit the characteristics of loss of polarity, reduced adhesion and increased mobility, which endow cells with the ability to invade and metastasize [10].

Multiple researches have shown that chemokines and chemokine receptors are involved in the growth, angiogenesis and distant metastasis of tumor cells in a variety of tumors [11]. C-X-C motif chemokine ligand 12 (CXCL12) is a member of the CXC chemokine subfamily, and its coding gene is located at 10q11.1 [12]. The C-X-C motif chemokine receptor 4 (CXCR4) is a specific receptor for CXCL12 [13]. Recent studies have found that C-X-C motif chemokine receptor (CXCR7) is the second receptor for CXCL12 [14]. Extracellular CXCL12 binds to CXCR4 or CXCR7 on the cell surface, and regulates cell function by activating various signal transduction pathways [15]. Studies have shown that the CXCL12-CXCR4/CXCR7 axis has multiple effects on tumors [16–18]. For example, CXCL12-CXCR4 has been found to promote the resistance of docetaxel in castration-resistant prostate cancer cells [19]. Xu et al. reported that CXCL12-CXCR7 axis promotes proliferation and metastasis of cervical cancer cells [20]. In gastric cancer, high-expressed CXCL12 and CXCR4 have been found to promote the migration, invasion and EMT of gastric cancer cells, and are closely related to the poor prognosis [21]. In addition, CXCL12 was found to enhance angiogenesis by activating CXCR7 [22]. In short, current studies have manifested that CXCL12/CXCR4/CXCR7 are important mediators for the malignant progression of tumors. However, their roles in EC are still unclear.

In this study, we aimed to investigate the regulatory role of CXCL12 and its receptor CXCR7 in the growth and metastasis of EC and the related molecular mechanism, so as to provide new targets for the treatment of EC. Our findings showed that CXCR7 was highly expressed in EC, and the knockdown of CXCR7 or CXCL12 inhibited the EMT process of EC cells. This research confirmed that the CXCL12/CXCR7 axis regulates EC cell proliferation, migration, invasion and EMT by activating the STAT3 pathway, thereby promoting the metastasis of EC.

## Methods

### Clinical samples

A total of 154 patients with esophageal squamous cell cancer (ESCC) who underwent surgery in Ningbo First Hospital from January 2013 to January 2017 were included in this study, including 119 males and 35 females with an average age of 62.2 ± 6.7. Among them, 12 cases were in Tumor Node Metastasis stage I, 86 cases in stage II, 52 cases in stage III and 4 cases in stage IV. None of them had received anti-tumor therapy (chemoradiotherapy, immunotherapy, etc.) before the surgery. Besides, 49 cases of normal para cancer esophageal squamous epithelium taken from a location greater than 5 cm from the edge of the cancer) were used as controls. All specimens were collected with informed consent of the patients and approved by the Ethics Committee of Ningbo First Hospital (No. 2022RS027).

### Cell culture

Eca109, CaEs-17 and HEEC cells were obtained from American Type Culture Collection. All cells were cultured and passaged with RPMI-1640 medium that containing 10% fetal bovine serum and 1% dual-antibodies at 37°C and 5% CO_2_. The reagents used for cell culture were purchased from Hyclone (USA).

### Quantitative real-time fluorescent Polymerase Chain Reaction (qRT-PCR)

The qRT-PCR was performed as previously described [23]. Total RNA of Eca109, CaEs-17 and HEEC cells was extracted using Trizol kit (Thermo Fisher, US). Then the total RNA was used as the template to synthesize cDNA using the reverse transcription kit (Biosharp, China). Next, the cDNA was used as template, and CXCR7 specific primers, SYBR Green qPCR Mix (Biosharp, China) and CFX fluorescence quantitative PCR instrument (Bio-RAD, US) were used to construct the qPCR system and carry out the reaction. The primers of CXCR7 and the internal reference GAPDH were as follows: CXCR7: Forward 5 ‘-GCAACTAGACAAGTTACGAACA-3’, Reverse 5 ‘-GCGAACTTAGCTATGACATCAG-3’; GAPDH: Forward 5 ‘-AGGCAATCAGTTAACATGACG-3’, Reverse 5 ‘-TAGGCAGACGATTACAGTAGC-3’. The primer sequences were synthesized by Synbio Technologies (China). The relative expression of CXCR7 mRNA was quantitatively analyzed by 2^−ΔΔCt^ method. The experiment was independently repeated for 3 times.

### Cell transfection

Eca109 and CaEs-17 cells were inoculated in a 6 cm dish and changed to serum-free medium when the confluence reached to 80%. The full-length CXCL12 cDNA open reading frame was inserted into pcDNA3.1 (+) vector (MiaolingBio, China) to obtain the recombinant plasmid pcDNA-CXCL12 that overexpressing CXCL12. pcDNA3.1 (+) empty vector (pcDNA-NC) was used as negative control (NC). si-CXCR7, si-CXCR4, Si-CXCL12 and their control were synthesized by Synbio Technologies (China). The sequences of the siRNAs were as follows: CXCR7 siRNA: 5’-CCGAUUACGACGUACUCGACTT-3’, CXCR4 siRNA: 5’-ACCUAUCGAAUUACGACUGTT-3’, CXCL12 siRNA: 5’-AUCAGACUUCAGACUCAGUATT-3’. pcDNA-CXCL12, pcDNA-NC, si-CXCR7, si-CXCR4, si-CXCL12 and si-NC were transfected with Turbofect (Invitrogen, USA) according to the directions. Cells were collected 48 h after transfection for other detections.

### Western blot

Eca109 and CaEs-17 cells were thoroughly lysed with RIPA lysate (Yeasen, China) on ice to extract the total protein. After the protein concentration was quantified, the same amount of proteins was subjected to sodium dodecyl sulfate – polyacrylamide gel electrophoresis, and the separated protein was transferred to polyvinylidene fluoride (PVDF) membrane. The membrane was then blocked with 1% bovine serum albumin (BSA), followed by incubated with primary antibodies overnight at 4°C. The next day, the PVDF was washed and then incubated with the second antibody for 1 h. Finally, ECL reagent (Thermofisher, USA) was dropped for developing. The relative expression level of target proteins was calculated on the basis of GAPDH. All antibodies were purchased from Abcam (UK), the antibodies and their dilution ratios were as follows: anti-CXCR7 (ab138509, 1:5000), anti-E-cadherin (ab40772, 1:30,000), anti-N-cadherin (ab18203, 1 µg/mL), anti-Vimentin (ab92547, 1:2000), anti-Snail (ab216347, 1:1000), anti-Slug (ab27568, 1:1000), anti-ERK1/2 (ab17942, 1:1000), anti-p-ERK1/2 (ab214362, 1:500), anti-AKT (ab8805, 1:500), anti-p-AKT (ab38449, 1:1000), anti-STAT3 (ab68153, 1:2000), anti-p-STAT3 (ab76315, 1:10,000), anti-α-tubulin (ab6046, 1:500), anti-GAPDH (ab9485, 1:2000), goat anti-rabbit IgG (ab6721, 1:10,000).

### Cell viability detection

Eca109 and CaEs-17 cells transfected with si-CXCR7 or pcDNA-CXCR7 or treated with AZD9150 were inoculated into a 96-well plate with about 1000 cells per well and 5 replicates were set for each group, with a volume of 200 μL per well. After about 6 h, 10 μL of CCK-8 reagent (MCE, USA) was injected and further incubated at 37°C for 2 h. The A_450 nm_ was measured by a microplate reader (CMaxPlus, MD, USA), and the cell viability was calculated.

### Wound healing assay

Eca109 and CaEs-17 cells were seeded in 6-well plates to spread the cells in a single layer in each well. After culturing overnight, sterile Tip was used to scratch the cells in each well, and the scratched cells were removed with PBS. Then cells were further cultured with the serum-free RPMI-1640. The width of scratches was measured and recorded at 0 h and 24 h after scratching. The cell mobility was then calculated.

### Transwell assay

First, the Matrigel (Corning, USA) was diluted with serum-free RPMI-1640 at a proportion of 3:1. Then 30 μL of the diluted Matrigel was evenly coated on the upper Transwell chamber and incubated at 4°C overnight. Eca109 and CaEs-17 cells were seeded into the upper chamber and cultured for 24 h. The cells invading to the lower chamber were fixed with 4% paraformaldehyde for 10 min. Then 0.1% crystal violet was used for staining for 30 min. Finally, the cells were photographed and counted.

### Immunofluorescence (IF) staining

First, sterile cell slides were placed at the bottom of 24-well plates, and Eca109 and CaEs-17 cells were seeded on the slides. Then the cell transfection was conducted. After 48 h, the medium was discarded, and cells were fixed with 500 μL pre-cooled fixative for 15 min. After rinsing, 500 μL of 0.5% Triton X-100 was added for cell permeabilization. Then the cells were blocked with 3% BSA. Primary antibodies anti-E-cadherin (ab40772, 1:500) and anti-vimentin (ab92547, 1:500) were added and incubated overnight at 4°C. Next day, fluorescent secondary antibody goat anti-rabbit IgG (ab150077, 1:500) was then incubated with cells at 37°C for 30 min in dark. After rinsing, the cell slides were taken out and dried, then pasted onto the cover glass that containing the mounting medium (with DAPI) (Mesgenbio, China), and the extra fluid was removed after light pressure. Images were taken by inverted fluorescence microscope (Ts2-FC, Nikon, Japan).

### Animal experiment

All mice were divided into 4 groups (n = 10) randomly: pcDNA-NC group, pcDNA-NC + AZD9150 group, pcDNA-CXCR7 group, pcDNA-CXCR7 + AZD9150 group. 1 × 10^6^ Eca109 or CaEs-17 cells over-expressed CXCR7 or NC were xenografted in the armpit of nude mice. STAT3 inhibitor AZD9150 (MCE, USA) was intratumorally injected into pcDNA-NC + AZD9150 group and pcDNA-CXCR7 + AZD9150 group. When the tumor volume of mice in pcDNA-NC group reached 100 mm^3^, all mice were sacrificed, the tumor, lung, liver and spleen of mice were removed for subsequent experiments.

### HE staining

The paraffin-embedded tissues were cut into 2 μm sections. After deparaffinage and rehydration, the sections were stained with hematoxylin and eosin successively, followed by gradient dehydration, and finally sealed with neutral balsam for microscopy.

### Immunohistochemical (IHC) assay

The paraffin sections were dewaxed and rehydrated, the following procedure was conducted according to previously described [24]. The sections were incubated with 3% H_2_O_2_ for 30 min to inactivate endogenous peroxidase. Then antigen retrieval solution (Beyotime, China) was used for antigen retrieval. After blocking with goat serum (Sigma, USA), anti-CXCR7 (AB72100, 10 μg/mL) or anti-Ki67 (AB243878, 1:500) was used to react with the sections overnight at 4°C. Goat anti-rabbit IgG (ab6721, 1:1000) was then incubated with the sections for 30 min. Afterward, the sections were washed with PBS and stained with diaminobenzidine, and then re-stained with hematoxylin. After dehydration, the sections were sealed and examined by microscope.

### Statistical analysis

SPSS 23.0 was used for data analysis, one-way ANOVA analysis was used for the measurement data among multiple groups, and the comparison between groups was performed by SNK analysis. Kruskal-Wallis H test was used for those with uneven variances. All data were expressed as mean ± standard deviation, and *P*< 0.05 was considered statistically significant.

## Results

### CXCR7 is highly expressed in EC

**Figure 1.**
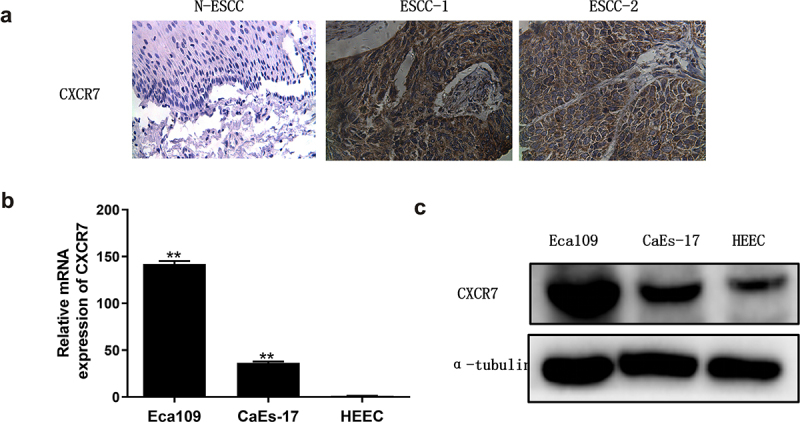
CXCR7 was highly expressed in EC. (a) The expression of CXCR7 in normal esophageal squamous epithelium and esophageal squamous cell cancer was detected by IHC. (b, c) The mRNA and protein level of CXCR7 in Eca-109, CaEs-17 and HEEC cells. **P < 0.01.

To determine the level of CXCR7 in EC, we analyzed it in tissue specimens of 154 patients with ESCC by IHC staining. In tumor tissues, CXCR7 was mainly expressed in the membrane and cytoplasm of cells (Figure 1a). According to the score, among the 154 cases, 116 cases (75.3%) were CXCR7 positive and 38 cases (24.7%) were CXCR7 negative. While the positive expression rate of CXCR7 in normal esophageal squamous epithelial tissues was only 6.1% (4/49), and all of them were weakly stained. The expression of CXCR7 in EC tissues was remarkably higher than that in normal tissues. In addition, mRNA and protein levels of CXCR7 in EC cell lines Eca109 and CaEs-17 were also significantly higher than that in HEEC, the human normal esophageal epithelial cell line, and the up-regulated expression of CXCR7 in Eca109 was particularly significant (Figure 1b and c). These results confirmed the high expression of CXCR7 in EC.

### Knockdown of CXCR7 inhibits EC cell proliferation, migration and invasion

**Figure 2.**
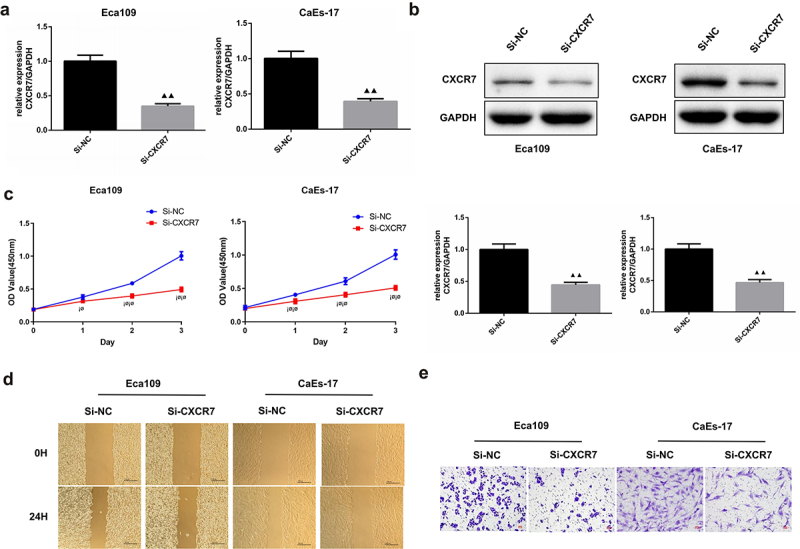
The effect of silencing CXCR7 on proliferation, migration and invasion of EC cells. (a, b) The transfection efficiency was confirmed by RT-PCR and western blot assays. (c) Cell viability of Eca-109 and CaEs-17 cells was assessed by CCK-8 assay. ^¡ø^*P* < 0.05, ^¡ø¡ø^*P* < 0.01. (d) Wound healing experiment was performed to evaluated the cell migration. Scale bar = 200 μm. (e) The invasion ability of the two cell lines was assessed by Transwell assay. Scale bar = 50 μm.

Next, we intended to explore the effect of CXCR7 on EC cells, so we knocked down the expression of CXCR7 in Eca109 and CaEs-17, respectively. The transfection efficiency was confirmed, the relative expression of CXCR7 in Eca109 and CAES-17 cells was reduced by more than 50% after transfection (Figure 2a and b). It was shown that compared with si-NC group, the cell viability of Eca109 and CaEs-17 cells in si-CXCR7 group was significantly decreased during 0–3 days (Figure 2c). The cell mobility of Eca109 and CaEs-17 cells in the si-NC group was 44.88% and 50.19%, respectively, while the mobility of Eca109 cells and CAES-17 cells decreased to 29.26% and 33.3% after knocking down CXCR7 (Figure 2d). This suggests that low expression of CXCR7 inhibits migration of EC cells. Moreover, the Transwell assay proved that the invasion ability of Eca109 and CaEs-17 cells was dramatically reduced after knocking down CXCR7 (Figure 2e). The above results verified that the knockdown CXCR7 inhibits the progression of EC cells.

### Effects of CXCR7 on EC cell morphology and EMT process

**Figure 3.**
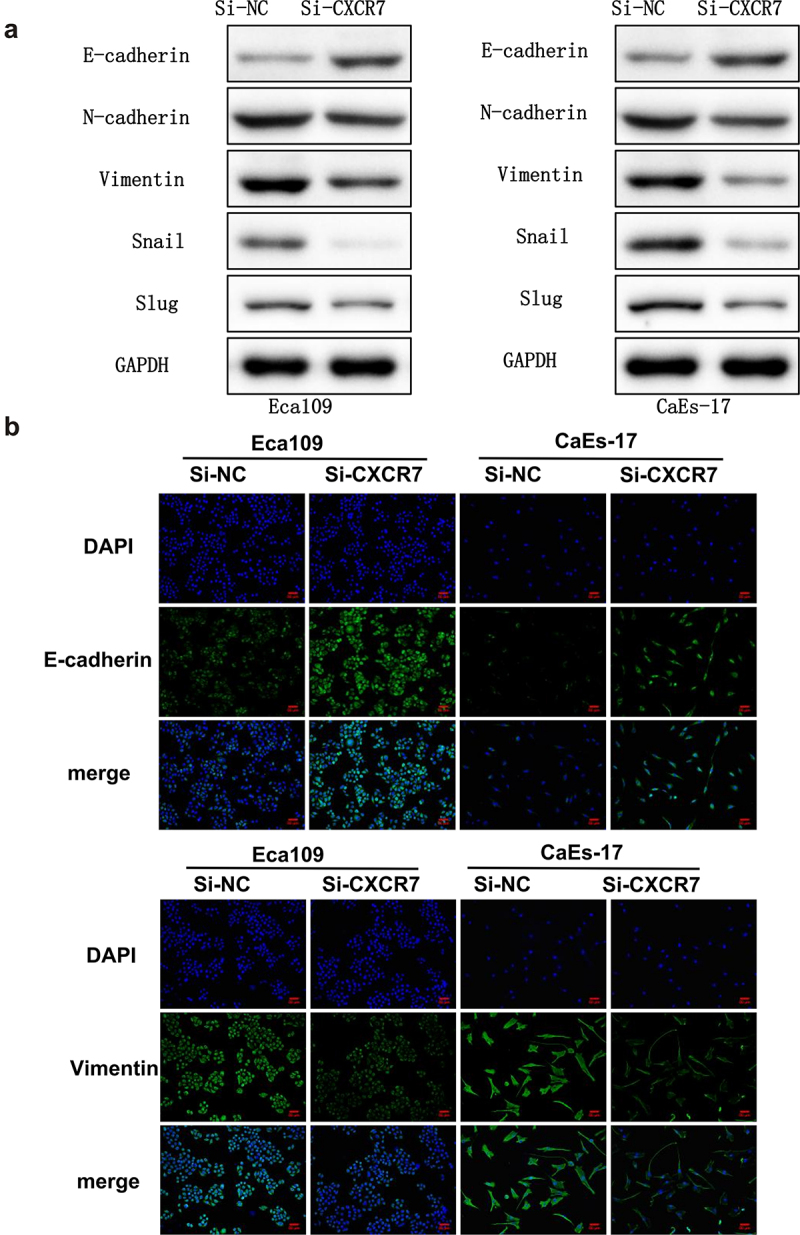
The effect of silencing CXCR7 on cell morphology and the expression of EMT-related proteins. (a) The expression of E-cadherin, N-cadherin, Vimentin, Snail and Slug was evaluated by Western blot. (b) The E-cadherin and Vimentin in Eca-109 and CaEs-17 cells were labeled by IF. Scale bar = 50 μm.

In order to elucidate the function of CXCR7 in the metastasis of EC, morphological changes and expression changes of EMT-related proteins in Eca109 and CaEs-17 cells after transfection of si-CXCR7 were studied. We assessed the level of EMT proteins in Eca109 and CaEs-17 cells. The expression of E-cadherin was obviously increased, while the expression of N-cadherin, Vimentin, Snail and Slug were markedly decreased compared with si-NC group (Figure 3a). Moreover, IF staining results also confirmed that the up-regulated expression of E-cadherin and the down-regulated Vimentin in EC cells of si-CXCR7 group (Figure 3b). These findings suggest that the knockdown of CXCR7 reverses the EMT process of EC cells.

### Impact of CXCL12-CXCR7 axis on the EMT process

**Figure 4.**
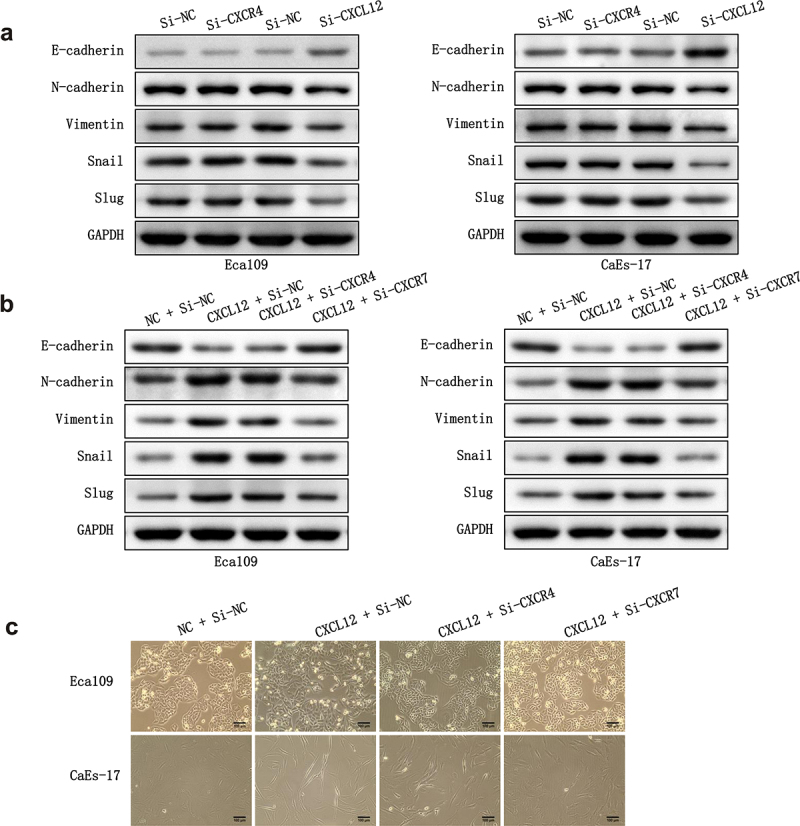
The effect of CXCR4/CXCR7/CXCL12 on EMT process. (a) The effect of silencing CXCR4 or CXCL12 on the expression EMT-related proteins. (b) The effect of overexpressing CXCL12 and knocking down CXCR4 or CXCR7 on the expression EMT-related proteins. (c) The effect of overexpressing CXCL12 and knocking down CXCR4 or CXCR7 on the mesenchymal morphologic changes of Eca-109 and CaEa-17 cells. Scale bar = 100 μm.

CXCL12 is a chemokine that binds to CXCR7, so we intended to know the regulatory effect of CXCL12-CXCR7 axis on the EMT of EC cells. In addition, as the classical receptor of CXCL12, whether CXCR4 interacts with CXCL12-CXCR7 to participate in EMT regulation is also unknown. Therefore, we knocked down CXCR4 and CXCL12 in Eca109 and CAES-17 cells, respectively. Subsequently, we overexpressed CXCL12 in the two EC cells, and then investigated whether knocking down CXCR4 or CXCR7 could reverse the effect of the overexpression of CXCL12 on EMT. After overexpressing CXCL12 alone, the level of E-cadherin in Eca109 and CAES-17 cells was lower than that in control group, while the levels of N-cadherin, Vimentin, Snail and Slug were higher. After overexpressing CXCL12 and knocking down CXCR4, the expression of EMT-related proteins did not change significantly when compared with pcDNA-CXCL12 + si-NC group. However, when CXCL12 was overexpressed and CXCR7 was knocked down, the expression level of EMT proteins was reversed (Figure 4b). Furthermore, we evaluated the influence of knocking down CXCR4 and CXCR7 on the mesenchymal morphologic transformation induced by overexpression of CXCL12. As shown in Figure 4c, compared with the pcDNA-NC + si-NC group, the intercellular contact between Eca109 cells in pcDNA-CXCL12 + si-NC group became loose, and the cells changed from pebble shape to elongated spindle shape. CaEs-17 cells also became very long and slender, and the intercellular contact was basically lost. The mesenchymal morphology of EC cells in pcDNA-CXCL12 + si-CXCR4 group was almost no change compared with the pcDNA-CXCL12 + si-NC group. While after overexpressing CXCL12 and knocking down CXCR7, the mesenchymal morphology of EC cells was remarkably reversed when compared with pcDNA-CXCL12 + si-NC group. These results confirm that inhibition of CXCL12-CXCR7 axis is beneficial to inhibit EMT, while CXCR4 is not involved in this regulation process.

### CXCL12-CXCR7 activates the STAT3 pathway

**Figure 5.**
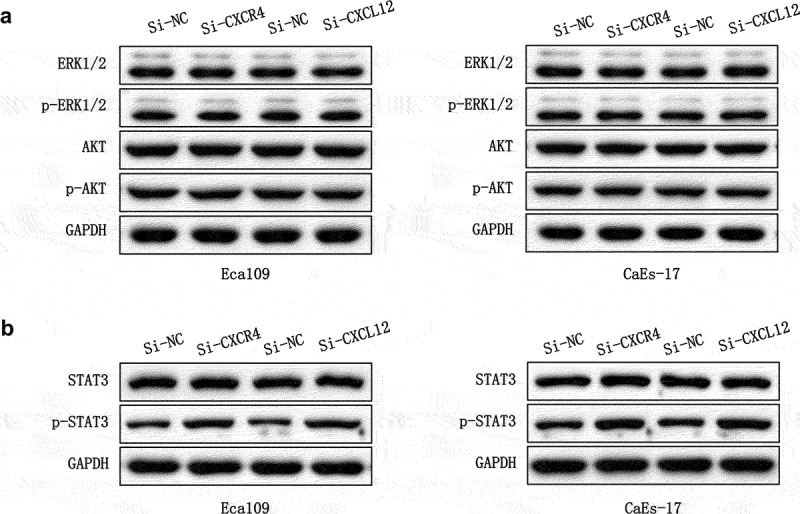
Activation of downstream signaling pathways by CXCR7/CXCL12. (a) The effect of overexpression CXCR7 or CXCL12 on the expression of ERK1/2, p- ERK1/2, AKT and p-AKT. (b) The effect of overexpression CXCR7 or CXCL12 on the expression of STAT3 and p-STAT3.

To further clarify the mechanism by which CXCL12-CXCR7 axis regulates EMT, we examined the activation of possible downstream signaling pathways. CXCR7 and CXCL12 were overexpressed in Eca109 and CAES-17 cells, respectively. The expression of ERK/Akt pathway proteins and their phosphorylated proteins were determined by Western blot, and the results showed no significant changes in the expressions of ERK1/2, p-ERK1/2, Akt and p-Akt (Figure 5a). Next, we detected the effect of CXCL12-CXCR7 on the STAT3 pathway, it was found that whether the overexpression of CXCR7 or CXCL12 significantly upregulated the level of p-STAT3 (Figure 5b). This indicates that CXCL12-CXCR7 activates the STAT3 pathway.

### Inhibition of STAT3 reduces the impact of CXCL12-CXCR7 on EC progression

**Figure 6.**
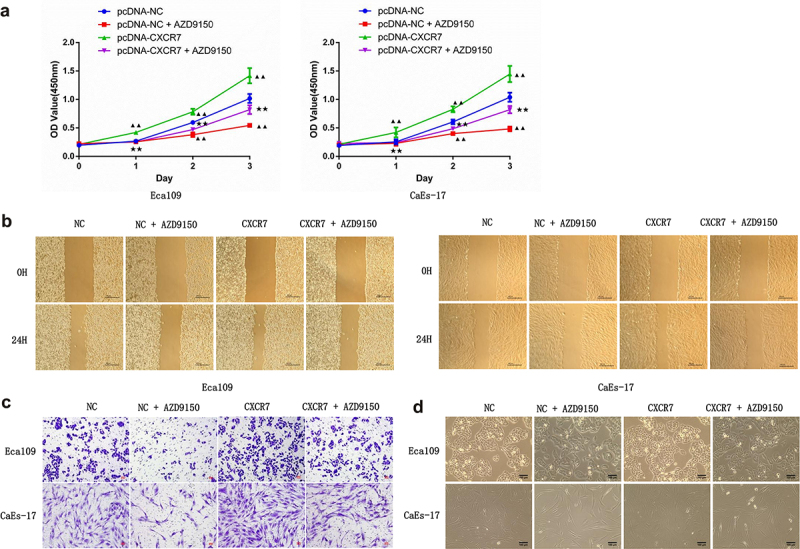
STAT3 inhibitor attenuated the effects of CXCR7/CXCL12 on proliferation, migration, invasion, and mesenchymal morphology of EC cells. (a) CCK-8 assay was performed to detected the proliferation of cells in pcDNA-NC, pcDNA-NC + AZD9150, pcDNA-CXCR7 and pcDNA-CXCR7 + AZD9150 groups. ^▲▲^*P* < 0.01 vs pcDNA-NC group. ^★★^*P* < 0.01 vs pcDNA-CXCR7 group. The migration and invasion ability of EC cells were detected by wound healing (b) and Transwell assay (c), respectively. Scale bar = 100 μm. (d) The mesenchymal morphologic changes of EC cells in the four groups. Scale bar = 100 μm.

Although we have identified the activation role of CXCL12-CXCR7 axis on STAT3 pathway, whether changes in this pathway affect the progression of EC remains to be studied. To this end, STAT3 inhibitor AZD9150 was added to the culture medium of Eca109 and CAES-17 cells transfected with pcDNA-NC or pcDNA-CXCR7. First, the transfection efficiency of pcDNA-CXCR7 was confirmed (Figure 6a). The cell viability of EC cells in the pcDNA-NC + AZD9150 group was significantly decreased, and significantly enhanced in pcDNA-CXCR7 group compared with the pcDNA-NC group. However, it was decreased when the co-existence of pcDNA-CXCR7 and AZD9150 (Figure 6b). The similar tendency was observed in the detection of migration and proliferation of EC cells in each group (Figure 6c and d), that is, AZD9150 reversed the effect of pcDNA-CXCR7. In addition, cell morphology of each group was observed. AZD9150 inhibited the transition of EC cells to mesenchymal morphology, and pcDNA-CXCR7 promoted the mesenchymal morphology transition of EC cells. However, when CXCR7 was overexpressed and AZD9150 was added, the number of EC cells with mesenchymal morphology decreased (Figure 6e). These results indicate that inhibition of STAT3 can weaken the influence of CXCL12-CXCR7 axis on EC progression.

### *Effect of CXCL12-CXCR7-STAT3 axis on EC progression* in vivo

**Figure 7.**
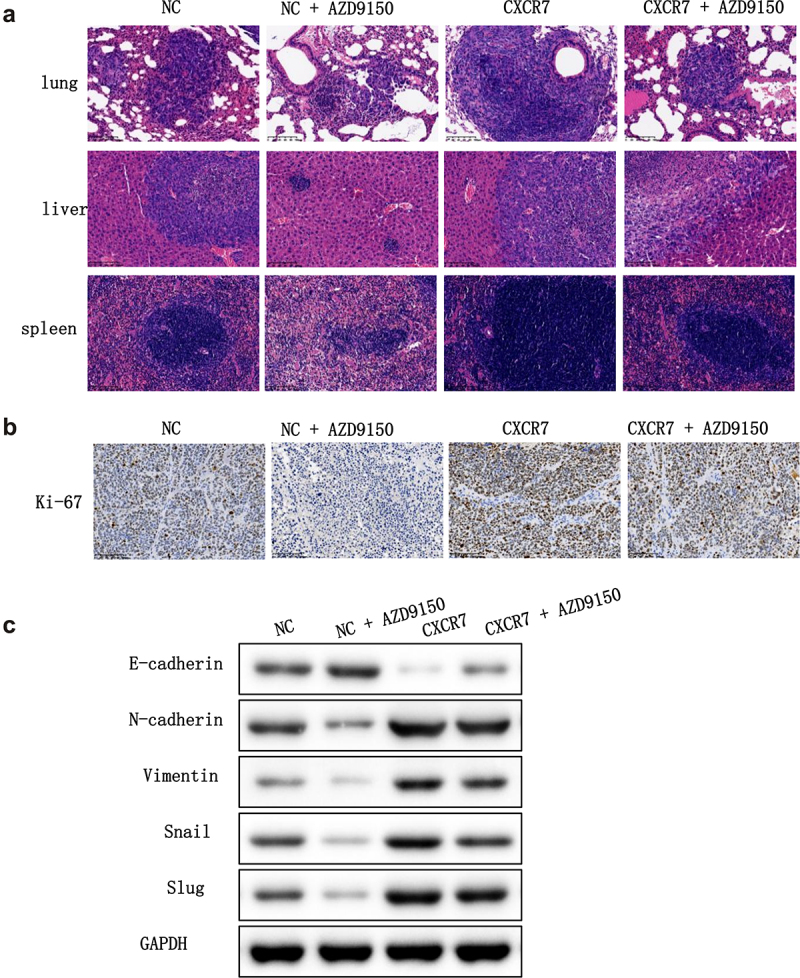
The effect of CXCR7/CXCL12/STAT3 axis on the growth and metastasis of EC cells in vivo. (a) HE staining was conducted to observe the metastasis of cancer cells in lung, liver and spleen. (b) Expression of Ki-67 in tumor tissues was stained by IHC. (c) The expression of EMT-related proteins in tumor tissues was detected.

Next, we intended to verify the effect of the CXCL12-CXCR7-STAT3 axis on EC progression in vivo. To this end, BALB/c nude mice were separated into four groups: pcDNA-NC, pcDNA-NC + AZD9150, pcDNA-CXCR7, and pcDNA-CXCR7 + AZD9150, and were treated accordingly. HE staining results showed that liver, lung and spleen in pcDNA-CXCR7 group were seriously infiltrated by tumor cells, and the score was the highest. The infiltration degree of liver, lung and spleen in pcDNA-NC group and pcDNA-CXCR7 + AZD9150 group was reduced. The infiltration degree of pcDNA-NC+AZD9150 group was the least, and the score was the lowest (Figure 7a). Ki-67 staining of tumor tissues by IHC showed that the number of positive cells in pcDNA-NC+AZD9150 group was the least, and it was the most in pcDNA-CXCR7 group. But the number of positive cells in pcDNA-CXCR7+ AZD9150 group was dramatically reduced (Figure 7b). Finally, we tested the level of EMT-related proteins in tumor tissues, and the results showed that AZD9150 reversed the effect of pcDNA-CXCR7 on the expression of EMT-related proteins (Figure 7c). Overall, these results suggest that inhibition of the STAT3 pathway in the xenograft mouse model suppresses the promotion effect of CXCL12-CXCR7 axis on EC progression.

## Discussion

EC is a malignant tumor that is prone to metastasis [25]. The spread and metastasis of tumor cells are very complicated, including abnormal apoptosis, migration and growth of tumor cells, tumor infiltration and immune escape caused by the changes in expression of specific molecules [26]. It is known that specific chemokine receptors are expressed on the surface of tumor cells, while the corresponding chemokines are highly expressed in sites that prone to metastasis [11]. Chemokines have chemotaxis to tumor cells, thus inducing targeted metastasis of tumor cells [27]. A great deal of studies has shown that the interaction of chemokines and their receptors is involved in the invasion and metastasis of various tumors [23,28,29].

CXCR7 is a member of the G-protein-coupled receptor superfamily and consists of 362 amino acids [30]. CXCR7 is highly expressed in many malignant tumor cells, such as lung, breast and cervical cancer [20,31,32]. In this study, we found that CXCR7 was highly expressed in esophageal squamous cell cancer tissues and esophageal cell lines Eca109 and CaEs-17. And the knockdown of CXCR7 inhibited the proliferation, migration, invasion and EMT process of EC cells. These suggest that CXCR7 plays a crucial regulatory role in the development and metastasis of EC. CXCR7 is another receptor that binds to CXCL12 besides CXCR4, and CXCR7 has a higher affinity with CXCL12 [18]. Existing studies have shown that the CXCL12-CXCR7 axis plays a biological effect similar to that of CXCL12-CXCR4 axis in promoting tumor cell proliferation, anti-apoptosis and tumor-related angiogenesis [15]. Recent studies have shown that CXCR7 can form a dimer with CXCR4 to act on the G protein coupling pathway after binding to CXCL12 [33]. However, some studies have found that CXCR7 can mediate the migration of neural progenitor cells to CXCL12 independently of CXCR4 [34]. In addition, the suppression of CXCR7 can inhibit the progression of cervical cancer cells induced by CXCL12, but CXCR4 is not involved in these processes [35]. Therefore, the signal pathways mediated by CXCR7 may be different from the typical chemokine pathways mediated by CXCR4. Here, we found that knocking down CXCR4 had little effect on EMT-related protein expression levels and the mesenchymal morphologic transformation of Eca109 and CaEs-17 cells. Besides, knocking down CXCR7 reversed the aggravated EMT induced by overexpression of CXCL12, but knocking down CXCR4 has no such effect. These indicate that CXCL12/CXCR7 but not CXCL12/CXCR4 is involved in regulating the metastasis of EC. Xin et al. also found that CXCR7/CXCL12 is closely associated with lymph node and liver metastasis in gastric cancer [36]. However, the specific signaling pathway involved in the combination of CXCR7 and CXCL12 has not yet been clarified, and further studies are needed.

Song et al. found that up-regulating CXCL12/CXCR4 in vascular endothelial cells activates MAPK/ERK and PI3K/AKT pathways, thereby accelerating angiogenesis in cancer tissues [24]. CXCR7 has also been found to regulate cell growth and angiogenesis in colon cancer by activating AKT and ERK pathways [37]. However, in this study, we were surprised to find that overexpression of either CXCR7 or CXCL12 did not change the expression levels of phosphorylated ERK and Akt proteins in Eca109 and CaEs-17 cells. This indicates that the CXCR7/CXCL12 axis does not activate ERK and AKT pathways in EC. Dramatically, we found that overexpression of CXCR7 or CXCL12 increased the expression of p-STAT3, and inhibition of STAT3 pathway weakened the facilitation of CXCR7/CXCL12 axis in proliferation, migration, invasion and EMT of EC cells. Furthermore, in nude mice xenografted with Eca109 and CaEs-17 cells, AZD9150, the generation 2.5 antisense oligonucleotide inhibitor of STAT3, was observed to inhibit tumor proliferation and liver and lung metastasis. AZD9150 also reversed the accelerated tumor proliferation and metastasis induced by CXCR7 overexpression. These demonstrate that the CXCR7/CXCL12 axis promotes tumor growth and metastasis by activating the STAT3 pathway in EC. Similarly, Yang et al. found that the suppression of CXCR7 in acute T lymphoblastic leukemia inhibits the activation of the STAT3 pathway, thus effectively controlling tumor progression [38]. The silencing of CXCR7 also inhibits the migration and invasion of tumor endothelial cells from hepatocellular cancer by inhibiting STAT3 [39]. In gastric cancer, CXCR7 was found to promote cancer progression through the STAT3/c-Myc pathway [40].

Collectively, our study confirmed that the level of CXCR7 was up-regulated in EC, knock-down of CXCR7 and CXCL12 inhibited the EMT of EC cells, while knock-down of CXCR4 had little effect on the level of EMT-related proteins and changes in mesenchymal morphology of cells. Furthermore, we demonstrated that the CXCL12/CXCR7 axis regulates proliferation, migration, invasion, and EMT of EC cells through the STAT3 pathway, thereby promoting the metastasis of EC.

## Conclusion

In conclusion, our findings confirmed that CXCR7 is overexpressed in EC, and CXCR7/CXCL12 axis promotes the growth and metastasis of EC by activating the STAT3 pathway. As a consequence, the intervention of the interaction between CXCR7 and CXCL12 is expected to be a new strategy for the anti-metastasis treatment of EC.
